# Application of the theory of para-social relationships for the analysis of people’s perceptions of indoor plants

**DOI:** 10.3389/fpsyg.2025.1533128

**Published:** 2025-06-18

**Authors:** Elizaveta Ivashkina, Oxana Mikhaylova

**Affiliations:** ^1^Faculty of Social Sciences, School of Sociology, Department for Social Institutions Analysis, HSE University, Moscow, Russia; ^2^Institute of Education, Centre for Modern Childhood Research, HSE University, Moscow, Russia

**Keywords:** para-social relationships, plants, people-plant interaction, indoor plants, qualitative research

## Abstract

**Introduction:**

Despite extensive research on the psychological and environmental benefits of houseplants, little is known about how individuals perceive and form emotional connections with them. This study addresses this gap by applying the theory of para-social relationships (PSRs)—traditionally used to analyze one-sided bonds with media figures—to human-plant interactions.

**Methods:**

Fifteen semi-structured, in-depth interviews were conducted with Russian-speaking university students who demonstrated close bonds with their indoor plants. The interviews explored the initiation, maintenance, and influencing factors of PSRs with plants, focusing on personal experiences and contextual variables.

**Results:**

Key findings reveal that exposure, homophily (perceived similarity), and contextual factors such as urban living and personal space are critical in fostering these relationships. Participants described engaging in regular care, observation, tactile interaction, and anthropomorphism to deepen their connections with plants. Five distinct types of human-plant relationships were identified: ownership, friendship, parenthood, sibling-like bonds, and neighborly relations, each varying in intensity and perceived plant agency.

**Discussion:**

The study highlights the therapeutic potential of PSRs with indoor plants in mitigating stress, enhancing emotional well-being, and providing companionship—particularly for students navigating transitional life stages or living in isolating environments like dormitories. These findings have broader implications for horticultural therapy, environmental psychology, and urban biophilic design. By extending PSR theory beyond human-media contexts to include non-human entities like plants, this research underscores the importance of fostering meaningful connections with nature to support mental health and promote sustainable living practices.

## Introduction

Humans have had a longstanding relationship with plants, with evidence of their cultivation dating back to ancient civilizations (Carey, 2023). This connection has been sustained throughout history, and plants have played a significant role in various aspects of human life, such as food, medicine, and cultural practices (Kaplan, 1987). Indoor plants have become increasingly popular in recent years, with many individuals turning to plant ownership to improve their well-being and indoor environment (Bratman et al., 2012). The psychological benefits of indoor plants have been well-documented in the extant literature. Studies suggest that they can enhance mood, reduce stress (Lohr et al., 1996), and improve cognitive functioning (Berman et al., 2008).

Interest in houseplants increased during the COVID-19 pandemic, as individuals started to spend more time at home and seek ways to create a comforting and relaxing atmosphere (Afacan, 2021) in an attempt to cope with the stress and uncertainty of the pandemic (Reis et al., 2020). The rise of “plant-fluencers” on social media platforms has also contributed to the increased interest in houseplants, providing inspiration and guidance on how to incorporate plants into indoor spaces (Carabelli, 2021).

Despite the widespread use and appreciation of indoor plants, little is known about the specific attitudes people hold toward them. This is where the theory of para-social relationships (PSRs) can provide a useful framework for analysis, providing a new lens through which to examine human-plant interactions. PSRs refer to the one-sided social bonds that individuals form with media personalities, fictional characters, or even non-human entities such as pets or objects (Brown et al., 2015). While the concept of PSRs has been widely researched in the context of media, little attention has been given to the potential for forming similar relationships with non-human entities such as indoor plants. This is surprising given that plants have been shown to possess characteristics that are associated with the formation of PSRs, such as the ability to elicit empathy, the capacity for reciprocity, and the potential for anthropomorphism (Calvo et al., 2020).

Understanding the possibility of PSRs with indoor plants is important as it can provide insight into the emotional and psychological implications of such relationships, though the lack of research on this topic makes that difficult. Studying students in this context is quite beneficial as this demographic group often faces conditions that are conducive to plant breeding, such as being exposed to stress in university (Ribeiro et al., 2018), living in densely populated places (Dai et al., 2010), and often having personal living conditions for the first time in their lives (Downing et al., 2007). Thus, the general research question of the following article is *“*How do university students form and maintain PSRs with indoor plants?” Sub-research questions include the following:

- SRQ1: How does the relationship with indoor plants begin and progress into a para-social one?
- SRQ2: What practices of para-social interactions and PSRs do students engage in with their houseplants?
- SRQ3: What types of PSRs with indoor plants can be distinguished based on the practices and attitudes people have toward their plants?
- SRQ4: What factors influence PSRs with indoor plants?

Examining students’ perceptions of and experiences with indoor plants, as well as the potential PSRs between them, holds significant importance. By analyzing how students interact with their plants, this study provides insights into how PSRs with indoor plants contribute to emotional well-being, stress reduction, and a sense of companionship in environments that might otherwise feel isolating, such as student dormitories (Shaikh and Deschamps, 2006). These findings can inform psychological support programs that incorporate plant care as a strategy for promoting well-being (Briggs et al., 2023).

Furthermore, educators can leverage these insights to explore innovative approaches to fostering environmental awareness and responsibility through plant-related curricula (Eugenio-Gozalbo et al., 2024). For researchers, understanding these dynamics broadens the scope of PSR theory by demonstrating its applicability beyond traditional human-media contexts and highlighting the broader implications of non-human companionship. Focusing on this specific population offers valuable perspectives on the psychological benefits, educational implications, and environmental considerations associated with indoor plants (Burke et al., 2022; Dünser et al., 2025; Stagg et al., 2025). By investigating the complexities of students’ PSRs with indoor plants, this study contributes to the existing body of literature and provides actionable knowledge for practitioners, educators, and researchers alike.

### PSR theory’s origin and associated constructs

The concept of para-social interaction was introduced in media studies by Horton and Wohl (1956), who studied how interactions between viewers of mass media and “media figures” (e.g., actors and celebrities) might result in a relationship in which the viewer responds as though in a conventional social relationship. A viewer’s response to a media performance in which they perceive the performer as an intimate conversational companion is referred to as a *para-social interaction (PSI)*. In this early work, Horton and Wohl also defined another concept, the *para-social relationship (PSR)*, which differs from the former in terms of length and depth of connection/bonding. While a PSR can last longer than a single viewing of a series episode, a PSI is limited to the time in which an episode is viewed.

During the development of para-social research, two main hypotheses have been proposed: the substitution hypothesis and the Panksepp–Jakobson hypothesis (Tukachinsky et al., 2020). The former (also dubbed the compensation/deficiency hypothesis) lies in the origin of the PSR concept: people look to the media to meet their emotional, cognitive, social, and other socio-psychological demands (Ruggiero, 2000). The substitution hypothesis has dominated PSR research for decades (Levy, 1979; Rubin et al., 1985; Tsao, 1996; Wang et al., 2008); however, it has also been challenged and criticized for oversimplifying the complex and dynamic nature of social relationships and the different motives for and functions of PSRs (Giles, 2002; Cohen, 2004; Lee and Watkins, 2016).

The Panksepp–Jakobson hypothesis avoids this critique, proposing that the human brain is wired to respond to social cues and interactions, even those mediated by technology or media (Jacobs and Willems, 2018). According to this hypothesis, when people engage in PSIs with media figures, they activate the same neural pathways involved in real-life social interactions, such as empathy, social reward, and emotional contagion (Stever, 2017). This can lead to the development of para-social bonds that mimic the emotional and social dynamics of genuine relationships, such as feelings of trust, loyalty, and intimacy. The current study uses this approach to examine potential PSRs with houseplants. Specifically, we turn to three concepts within the Panksepp–Jakobson hypothesis: homophily, exposure, and exposure context.

*Homophily*, the propensity to connect with those perceived as similar to oneself, is a significant factor influencing interpersonal dynamics (Fehr, 2008). Intriguingly, this principle extends to people’s preferences and perceptions concerning indoor plants, revealing its relevance in the context of human-plant relationships. Research has shown that individuals often gravitate toward indoor plants that bear visual or cultural resemblance to those plants they are already familiar with (Lohr and Pearson-Mims, 2006). Additionally, individuals may exhibit a proclivity to develop PSRs with indoor plants that they perceive as sharing personality traits akin to their own. The yet scientifically unexplored topic of homophily in relation to plants is evident in various online articles, where people create quizzes such as “The best plants for you based on your personality type” https://www.greenmatters.com/home/2018/06/25/Z1FtiGB/houseplant-personalities or write articles like “Here Are The 8 Best Houseplants For Every Personality” https://www.wellandgood.com/lifestyle/kinds-of-plants-personality. This illustrates the role of homophily in shaping the nature and strength of these human-plant connections. Importantly, the significance of homophily in the context of PSRs with indoor plants mirrors its impact on PSRs with media figures. Studies have demonstrated that individuals are more likely to form PSRs with media personalities they perceive as similar to themselves (Cohen, 2004). Similarly, homophily with indoor plants can engender a heightened sense of connection and attachment, potentially leading to more enduring and meaningful PSRs.

*Exposure*, another crucial concept in para-social research, mirrors quality time in typical relationships. Time investment is vital for relationships to evolve, with acquaintanceship transitioning to friendship after approximately 30 h, and close friendships requiring about 140 h (Hall, 2019). Shared activities during this time, like self-disclosure and humor, fulfill the human need for belonging (Hall et al., 2017). One key mechanism through which exposure fosters PSRs is the development of emotional investment. In media-based PSRs, audiences become emotionally invested in characters’ experiences, sharing their highs and lows. Similarly, with plants, this emotional investment emerges through ongoing care and maintenance routines. As individuals monitor their plants’ health, adjust their environment, and respond to changes, they cultivate a sense of responsibility and attachment that parallels the emotional investment observed in other forms of PSRs (Clayton and Meyers, 2015). In PSRs, audience exposure to media figures resembles shared time. It allows individuals to learn about and bond with media characters by virtually participating in their activities. This time investment, akin to building friendships, enhances commitment (Eyal and Dailey, 2012; Hall et al., 2017). Regarding PSRs with indoor plants, exposure significantly influences attitudes and emotions. Studies show that exposure to indoor plants improves mood, reduces stress, and boosts well-being (Lohr and Pearson-Mims, 2006; Ratcliffe et al., 2016). Positive feelings toward plants form the basis of PSRs, as individuals see them as comforting. Exposure also leads to perceiving plants as social beings, enabling anthropomorphism (Frijda, 1986). This fosters connection and a desire for regular interaction (Kaplan and Kaplan, 1989). Beyond general well-being, exposure to plants also facilitates a form of social learning, wherein individuals begin interpreting plants’ growth patterns and responses as forms of non-verbal feedback. Research suggests that individuals who frequently engage with plants are more likely to anthropomorphize them, attributing emotions or states of being based on observable cues such as wilting, new growth, or leaf positioning (Epley et al., 2007). This interpretative process—where plants’ reactions are perceived as indicative of “needs” or “preferences”—is central to the formation of PSRs, as it reinforces the perception of plants as social partners rather than static objects.

*Exposure context*, a crucial variable in understanding para-social relations with plants, deserves attention. Research has shown that para-social connections vary depending on the context. For example, interactive stimuli, like video games, tend to induce stronger connections than spectator media like videos (Tukachinsky, 2014). In PSRs with indoor plants, exposure takes various forms, from physical presence to social media. Previous studies have highlighted the positive impact of exposure to indoor plants. Their mere presence in workplaces and homes can boost mood, reduce stress, and enhance productivity (Bringslimark et al., 2009; Lohr and Pearson-Mims, 2006). However, passive exposure alone is insufficient for the development of PSRs—interaction is essential. Social media, for instance, provides a platform where plant owners can share their experiences, seek validation, and strengthen their attachment through communal discourse (Carabelli, **2021**). Studies on digital plant communities have demonstrated that plant keepers attribute meaning to their plants through shared narratives, framing plant care as a journey marked by both setbacks and achievements (Burke et al., 2022). This form of mediated interaction enables individuals to compare their relationships with plants to those of others, thereby deepening the personal significance of their own plants.

Additionally, the popularity of indoor plants on social media platforms has drawn individuals into plant cultivation more enthusiastically (Carabelli, 2021). Exposure to biophilic interior design, which acknowledges our innate connection with nature (biophilia), has been beneficial for reducing anxiety and stress. Those in biophilic environments experience better recovery outcomes from serious mental health issues, enhancing psychological, social, and physical well-being (Grinde and Patil, 2009). The mechanism by which these environmental factors contribute to PSRs extends beyond mere feel-good effects. Unlike other comforting stimuli, such as food or music, which primarily provide passive enjoyment, plants require a level of engagement that fosters a sense of mutual responsiveness. For example, when individuals water a plant and later observe signs of growth, they perceive a reciprocal interaction, reinforcing the idea of the plant as an entity with needs and responses. This feedback loop of action and reaction strengthens PSRs by mirroring the fundamental structure of social relationships, where investment leads to a perceived connection (Tam et al., 2013).

By unpacking these mechanisms—emotional investment, social learning, mediated interaction, and perceived reciprocity—it becomes clear that PSRs with plants are not merely the result of exposure alone but rather the result of repeated, meaningful engagement that fosters a perception of connection over time. These mechanisms work together by transforming routine care into emotionally significant rituals and by anthropomorphizing plant responsiveness, which creates a sense of mutual interaction. This context contributes to the development of robust para-social connections with indoor plants, fostering a sense of companionship and connectedness, two key components of PSRs (Schramm and Hartmann, 2008).

### Students and indoor plants in PSR research

In recent years, many scientists around the world have emphasized the importance of studying students with regard to people-plant relationships (Han, 2009; Daly et al., 2010; van den Bogerd et al., 2020). Students represent a unique population due to their diverse backgrounds, daily routines, and exposure to various environments, including educational institutions and living spaces such as dormitories and apartments. Research focusing on students’ perceptions of and experiences with indoor plants holds great importance for several reasons.

First, understanding students’ perspectives and experiences can provide valuable insights into the role of indoor plants as sources of psychological support and companionship in academic settings. Higher levels of stress and anxiety are commonly reported among students (Stallman, 2010), and having indoor plants may serve as a coping mechanism and contribute to their overall well-being. Exploring students’ PSRs with indoor plants can shed light on the potential psychological benefits and how these relationships contribute to their daily lives and academic experiences.

Additionally, sociological research has explored the influence of social norms, peer relationships, and group dynamics on students’ interactions with indoor plants and the development of PSRs. Research has demonstrated that social interactions and shared experiences related to indoor plants can strengthen social bonds among students and contribute to the formation of collective identities (Stapleton and Meier, 2022). For instance, students who participate in plant care communities—such as dormitory gardening clubs or online groups focused on plant care—engage in shared rituals, discussions, and collaborative problem-solving that foster a sense of belonging. These collective practices reinforce group identity by establishing common values (e.g., sustainability, mindfulness) and creating a shared language around plant care. Furthermore, the symbolic meaning attached to plants within a group—for example, gifting plants as tokens of friendship or using them to commemorate milestones like graduation—further solidifies these social connections. Over time, these shared experiences shape students’ sense of identity within these communities, transforming plant-related interactions from individual activities into integral components of a broader social fabric. Such insights highlight the importance of social factors in shaping the motivations, practices, and intensity of students’ PSRs with indoor plants.

Moreover, studying students’ PSRs with indoor plants can contribute to the broader field of environmental psychology. Research has shown that exposure to natural elements, such as indoor plants, can have positive effects on individuals’ psychological well-being and cognitive functioning (Kaplan, 1995; Bringslimark et al., 2009). Exploring the para-social dimensions of these relationships provides a unique perspective on how individuals interact with and derive benefits from indoor plants. This increases the understanding of the psychological and emotional connections between humans and nature.

## Method

### Data gathering methods

The study employed a qualitative research design to explore the nuanced and subjective experiences of individuals engaging in relationships with houseplants, which would be challenging to capture through quantitative methods. Data were collected through semi-structured, in-depth interviews with students who owned and cared for indoor plants. This approach was chosen for its flexibility, as it allowed participants to narrate their experiences in their own words while enabling the researcher to probe deeper into emergent themes.

Overall, 15 interviews were included in the empirical foundation of the study. They were conducted in April and May of 2023. The volume of cases is explained by the challenges in finding a sample due to the low level of reflection on everyday practices that indicate close relationships with houseplants. Additionally, the number of interviews collected is justified by the concept of minimizing repetitions and determining the optimal ratio between time and resources expended, as discussed by Steinberg (2014). All interviews were conducted in Russian and took place in an online format using the video conferencing platform Zoom, which, when possible, allowed participants to show their houseplants in the frame while they discussed them.

In addition, it is important to note that most of the participants were in Moscow at the time of the interview; however, territorial differentiation was still present. Three respondents were not living in Russia at the time of the interview: one lived in Istanbul, Turkey; one lived in Brussels, Belgium; and the third lived in Kampala, Uganda. Four respondents lived in a dormitory, three lived in their family home with their parents, two lived alone in their own space, and the rest lived with roommates or partners.

The guide for the interview was compiled by taking into account the spectrum of people-plant interactions proposed by Haller et al. (2019) and factors of PSRs introduced in Tukachinsky et al. (2020). At the initial stage, two pilot interviews were conducted, which showed that in general the guide was well compiled but required small revisions. Both pilot interviews were conducted on the Zoom video conferencing platform and lasted about 40 min.

### Sampling strategy and final empirical base

The main criteria for recruiting participants were: (1) being a university student and (2) demonstrating a close relationship with houseplants. For the purposes of this study, a “close relationship with houseplants” was operationalized through specific behavioral and emotional indicators. These indicators included regularly engaging in plant care routines (e.g., watering, repotting, pruning), attributing social or emotional significance to their plants (e.g., naming them, talking to them), and expressing a sense of attachment or responsibility toward their well-being. During the recruitment process, potential participants were asked preliminary questions about their interactions with houseplants to ensure they met these criteria.

The study employed theoretical sampling with a “snowball” selection logic. Theoretical sampling focuses on studying the properties of a specific phenomenon rather than a particular social group, allowing for the expansion of empirical data to guide theory development (Steinberg, 2014, p. 44). In this study, the phenomenon under investigation is the parasocial relationship (PSR) individuals form with indoor plants. The “snowball” method enabled the researcher to identify and recruit respondents who possessed the necessary insights to explore this phenomenon.

The method of participant selection was closely aligned with the utilization of network structures to access an empirical field that is challenging to reach. Given that parasocial relationships with plants are not a widely recognized phenomenon, identifying participants required leveraging existing networks of plant enthusiasts. Consequently, the initial entry points were acquaintances who provided access to individuals with specific relationships to their indoor plants that could be characterized as parasocial. This strategy ensured that the sample included participants who were both aware of and capable of articulating their plant-related experiences. A list of respondents and their main characteristics is provided in Appendix 2: Table 1.

### Data analysis

In this study, the thematic analysis method developed by Virginia Braun and Clarke (2006) was employed to analyze the data collected from the interviews. Thematic analysis is a widely used qualitative research approach that allows for the identification and interpretation of patterns, themes, and meanings within the data (Braun and Clarke, 2006). The decision to use thematic analysis in this study is based on its flexibility and adaptability, making it possible to capture the rich and diverse experiences and perspectives of the participants. By employing this approach, the research aims to identify recurring themes and patterns related to the practices, motivations, and intensity of participants’ PSRs with their indoor plants.

In the thematic analysis, the coding logic was deductive, with topics predetermined by analyzing the existing literature. The algorithm for the analysis procedure was based on Bryman (2016). First, the interview materials were carefully read. During this phase, initial observations were documented, and key phrases were highlighted to identify potential codes. The second step involved open coding of the data, where specific excerpts were labeled with descriptive codes that captured the meaning conveyed by participants. These codes were then grouped into themes, which were evaluated and named. Subsequently, connections between the themes were established, and key research insights were articulated during the analysis, creating a coherent narrative about the data. To ensure the reliability of the coding process, selected transcripts were reviewed by a second researcher to cross-check coding consistency.

## Results

### The beginning of the PSR with houseplants

Most participants’ history with indoor plants began in early childhood, specifically due to their mothers’ interest in plants. For many respondents, it was common to have plants in their living spaces during childhood, not only in shared spaces like kitchens and hallways but also in their bedrooms. This was only sometimes perceived positively. It was surprising how many of those who love houseplants today noted that as a child they were highly irritated by plants in their personal space: “It really annoyed me to have them in my room, to the point that I just did not like them at all. They created an extra burden because I had to take care of them” (6KR).

However, familial interest in plant growth was only sometimes passed down; there were repeated instances in the sample in which getting a plant and starting to care for it was solely the initiative of the respondent. Some of these students already had small quantities of plants in their childhood home before their own interest in plants was developed, but the care that the adults provided was inadequate. Sometimes these initial family plants, which were not actively cared for, became the first “wards” of respondents.

The change in patterns of houseplant ownership primarily showed when participants moved into a new space, as plants were a way to make the space “their own.” This motive was voiced by respondents in different life circumstances: some had changed living arrangements within their parents’ residence as their older siblings moved out and the respondent could manage their room as they wanted; others moved to another city or even country during their studies and started living in a dorm that they wanted to personalize; some respondents moved into new apartments, living on their own for the first time; and some moved in with a partner or a roommate. In all these situations, getting a plant was almost always considered to be an obligatory part of the process.

### Plant interaction practices and narratives to legitimize them

In the context of plant interaction practices, care was most often discussed. This refers to the specific routine with the plant—watering, misting, replanting, fertilizing, and other care practices.

For some of the participants, the plant routine was a pleasant pastime that provided a chance to ground, slow down, and “get out of the capitalist life, out of the city where everything is gray and cynical” (8ND). For others, it was more of an obligation associated with the responsibility accepted when the plant was purchased. As one respondent said, “We are responsible for what we plant” (9SG).

Some respondents shared caring for a plant changed their attitude toward the particular plant and plants in general. One respondent said that she had bad experiences with plants and developed the reputation of a “plant killer.” Though she still did not trust herself with plants, at the time of the interview, she had been successful in taking care of a large number of new plants for quite a while. Other respondents, who also faced difficulties when caring for plants but had positive outcomes, shared their stories with positivity, talking about “saving” their plants with pride and confidence:

This one tree of mine was dying; I do not know what was wrong with it. Nevertheless, we still did everything to save it. I found some wild rituals on the Internet: wet it for 2 h, spray it with this and that, and plant it in fresh soil. And oh, hallelujah! It stopped dying. That is my biggest accomplishment as a gardener so far; I have never felt better about myself. (1LS).

Another frequently mentioned practice was observation. In the care context, observation was often voiced as a part of the routine or direct addition to it: “It is difficult to water the flowers without looking at them” (9SG). For some respondents, it was a separate practice, and observation was seen as an opportunity to wind down or switch off while studying.

It is interesting to note the care practices that went beyond the basics. For example, observation was seen not only as a preventative measure of diseases that could otherwise go unnoticed, but also as a “demand” from the plant for its more vigorous growth: “I have a strong feeling that orchids need to be looked at, to be in a prominent place, and to be admired, as if they demand it from me” (11KR). Furthermore, other participants talked about using their personal things in caring for plants; for example, a girl growing potatoes mentioned that she regularly wraps her plants with her scarf for fear that they might freeze at night, and another respondent waters his plants from the same bottle that he drinks, “because my plants deserve no less than me” (9SG).

Interaction practices other than caring for plants were less frequently voiced but were still present in the narratives. First, respondents shared the importance of tactile interaction; simply touching the leaves periodically or digging in the soil was a calming practice, allowing one to slow down and ground themselves: “If something blooms or a bud appears, it feels good to touch it because it connects me with reality” (7AA).

Another type of interaction was recreation. This includes the abovementioned aspects of observation, care, and other forms of slow and quiet interaction with indoor plants. For example, one of the respondents talked about her tradition of organizing a “bath day” for her plants on which she bathes them in the shower; on such days, all other activities are put aside, and all attention is given to the plants. Despite some physical activity, she felt rested and recovered at the end of the day.

### Types of people–plant relationships

In general, five types of attitudes define relationships with plants: ownership, friendship, parenthood, and sibling and neighborly relationships. These attitudes differ in terms of how an individual perceives the agency of the plant, with no agency when houseplants are viewed as possessions and with an increasing level of agency depending on the perception of a plant as a child, a sibling, a neighbor, or a friend. The intensity of PSRs with indoor plants varies based on the type of attitude one holds and from plant to plant.

The first type of attitude, ownership, was characterized by a slight emotional connection with a plant; it served fundamental functions for the respondents but nothing more. At the other end of the spectrum was the parental attitude toward plants, with some respondents referring to them as their “babies” that need constant and sensitive care. In these cases, the plant was viewed to have less agency and depend on its keeper, and the bond that occurred between the owner and the plant was very powerful and enduring.

Those who had a sibling-type relationship with their plants perceived them as independent beings whose growth depended on them in some way. The comparison of plants to younger siblings was often used here; in the same way human relationships are built in a family, positive attention from older siblings improves the younger sibling’s quality of life.

Those who perceived plants as neighbors discussed plant independence more frequently, but at the same time, the aspect of equivalence in this relationship was important; each side of the “arrangement” contributed to the life of the other. In this view, the human meets the needs of the plant by taking care of it, and in return, the plant decorates the space and produces more oxygen. Various contributions of plants were mentioned, from producing a pleasant smell in the space to keeping a person company when they are lonely. This is what one of the respondents said about his plants: “We are neighbors in the same space who are engaged in a common cause—trying to survive on this planet” (9SG).

The last attitude is the view that plants are friends; here, the idea of plants being equal to people was made absolute: “It is definitely a friendship with creatures equal to you, just encased in another shell from which they cannot do anything for themselves” (11KR).

As mentioned above, many factors influence people’s relationships with their plants. The relationship’s intensity changes over different periods, so respondents voiced narratives showing that all plants occupied different positions on the spectrum at any given moment and one plant could go from one category to another more than once.

### Factors influencing people–plant interactions and relationships

#### Space

Space was an essential aspect of plant keeping, as a change in living situation was a common reason to buy a plant. Finding oneself in a new space was usually accompanied by the idea of getting a houseplant to create feelings of coziness and livability. This was especially important for students who were moving into a dormitory:

There were bare walls everywhere, almost as if you were in a hospital. It was impossible to live like that, so the first thing I did when I moved in was to make sure there was at least some kind of plant. (5NY).

How the space was arranged with the plant was often as important as the attitude toward the plant. In this context, respondents often mentioned that they had a “hierarchy” among plants, which influenced their placement in the space. Personal judgments about where one wanted to see which plants were taken into account; respondents told the researcher that “ugly plants” were often placed in the back row because they did not want to throw them away, but did not want to look at them in the front row either. Furthermore, the respondent could perceive the feelings of the plant regarding, for example, an undesirable location or the loneliness of the plant, which should be corrected: “This palm tree should be put next to some other flowers because I feel like they are getting lonely” (11KR).

#### Moving

Moving often marked the start of houseplant care, but it also revealed interesting aspects of PSRs. Respondents who had formed bonds with their plants while living elsewhere usually chose the easiest-to-transport plants during relocation. Practical considerations, such as quarantine requirements and high transportation costs influenced their choices. However, some respondents creatively navigated shipping rules by transporting plants unofficially, without soil and pots.

Furthermore, individuals were hesitant to subject their plants to the moving process due to the stress it imposed on both them and the plants. One participant expressed this concern, stating: “My most cherished plants weigh about 10 kilograms, pot included. Unfortunately, pots cannot be moved, and I cannot expose my beloved plants to such stress” (4DH).

When moving, plants also helped individuals to settle in the new residence and became the first “friends” with whom one could, for example, talk in their native language, as one of the respondents shared. People who moved to a new country, where life was not established and everything was painfully unfamiliar, were usually in particular need of this: “I did not have friends and tight ties in the new place then, and through this routine with plants, I was trying to make a ritual through which I could rejoice in the little things” (13DS).

#### Big city life

Most of the participants in the sample lived in big cities, and several of them previously lived in much smaller towns. This allowed them to reflect on the influence big city life had on their perception and attitudes toward indoor plants. Some noted that despite the difference in lifestyle, their experience with indoor plants did not change drastically; others noted that moving to a large city made them interested in getting houseplants in the first place, as they were exposed to a gray, noisy and exhausting environment:

I actually lived in a village before that. A lot of nature, the view of the field and the forest from the window. I did not think about plants that much. Here, when the view from the window is the construction site and everything is so dull, I really wanted something positive. (10MP).

Almost all respondents mentioned that plants make life in the city more pleasant, helping to introduce a calm and slow routine into their daily lives that positively affected their mood and well-being. Some respondents even referred to houseplants as a full-fledged sanctuary of nature that can be reached “while lying in bed.” For others, the houseplants are more of an artificial piece of nature that is certainly nice to have around but do not compare with organic nature: “Rather, I feel the need to leave Moscow more often than having a patch of houseplants on the shelf.” (2VN).

#### Plants and other people

An essential aspect of the relationship between people and plants was including others in the process. When describing their history with plants, almost all respondents also talked about other people’s contributions. Whether parents influenced the desire to get a plant, an aunt had a favorite flower, or a friend gave a cutting, there were numerous stories of how others contributed to respondents’ interest in plants. New connections between people also emerged based on plants; for example, one respondent said that she and her neighbor sprouted beans and now share the responsibilities of caring for them. Another participant said her husband had given her a plant as a birthday present and immediately named it, although he had never been interested in plants, and it became a running family joke.

Plant owners also make assumptions about other people based on their attitudes toward plants. Several respondents said they considered the condition of a houseplant to be a reflection of what kind of person takes care of it (if they take care of it at all). Here is what a respondent said about how she perceives other people in the context of plants: “If this is a rude person who has no interest in life, no understanding of beauty, then he definitely will not grow an avocado from a seed” (5NY).

It was also intriguing to observe how associations between certain plants and specific people were formed. For example, one informant shared with the researcher that she never liked “grandma’s” flowers, such as violets, because her grandmother had too many of them. Respondents also recounted stories about their inability to understand why some people prefer certain flowers and the conclusions that can be drawn from this: “I am always amazed by people who like orchids. It could be a compatibility test; two people like orchids—they are a super couple.” (13DS).

#### Media

It is almost impossible to avoid consuming media in the modern world, even when it comes to the topic of plants. Some respondents noted that they developed an interest in plants while watching *Leon*. This movie was mentioned more than once, and several people named the Aglaonema—the plant owned by the main character—as their favorite type of plant. One participant shared a story about how he had been waiting for years for his parents to allow him to get a cactus. This desire stemmed from watching an episode of Smeshariki (a popular Russian children’s cartoon series) as a child, in which one of the characters, the Hedgehog, had a cactus collection. Feeling a connection to this character, the respondent believed that getting a cactus would manifest this connection.

Some respondents became attracted to the idea of having plants after looking at “aesthetic pictures on the Internet*”* and realized that they wanted to create a “cottagecore” aesthetic in their living space. There was also a respondent who watched shows that had plants in the background and played a game with his friends in which they guessed what specific plants were in the frame.

However, some participants refused to consume plant-related content because blogs and social media posts on the topic made them feel anxious about the way they treat their plants. They emphasized the relative lack of care they gave their plants and compared themselves to “bad mothers.” However, they were much more positive about personal exchanges on social media, talking about their membership in several group chats dedicated to discussing houseplants. One such group chat was “My Ugly Plant,” where people share photos of their plants that are not particularly aesthetically pleasing. This was seen as a rewarding and motivating experience because people shared not only their successes but also the challenges of caring for plants.

For respondents who created social media content, plants often served as a pleasant addition to their posts. One participant, a microblogger, shared: “My top comments were always either ‘you look like Kendall Jenner’ or ‘what plants are those,’ and that is good for the numbers” (2VN). This attention to her plants was gratifying, but not everyone’s experience with posting about plants on social networks was as enjoyable.

Another respondent explained: “I do not specifically post my plants anymore on social media. It’s such a silly prejudice, of course, but it’s there because I have the experience of having my flower jinxed, and I do not want that to happen again” (12KS).

## Discussion

The purpose of this article was to study how students form and maintain PSRs with indoor plants. The study partially confirmed that PSI practices and relationship formation would primarily be shaped by individuals’ initial exposure to houseplants. Typically, the first exposure to indoor plants occurred within the family home where close relatives owned and cultivated them. Children in such settings often began participating in plant care and assuming responsibilities. However, many participants recalled not being interested in this activity during their early years and viewed it as burdensome. Over time, as students developed an interest in houseplants, their family environment became a significant source of information and support, influencing their attitudes and practices toward indoor plants. This finding aligns with existing literature on people-plant interactions, which underscores the impact of family history and the social-historical context of human perceptions and attitudes toward plants (Del Sesto, 2020).

Another notable finding was that during periods of separation, particularly due to changes in living situations, individuals used plants as a means to personalize their new spaces. Research on biophilic design has emphasized the benefits of incorporating plants into living spaces, as they contribute to a calming and refreshing atmosphere that enhances overall well-being (Ryan et al., 2014). This practice is particularly pronounced in highly urbanized areas, as confirmed in this study. Many participants relocated from smaller towns to metropolitan areas during their studies and acquired plants to mitigate the stress of urban living, thereby creating a cozier and more pleasant living environment. Additionally, the study revealed the reciprocal nature of plant acquisition, with several participants indicating that purchasing a plant was often their last resort. Instead, they frequently received or were gifted plants by acquaintances, friends, or relatives. The practice of gifting and exchanging plants gained prominence when it was introduced by other plant enthusiasts. This pattern of plant acquisition reflects the multifaceted nature of gift-giving, which can be both generous and strategic (Khalil, 1997). People often give plants as thoughtful gestures, but this practice can also benefit their own indoor plant situations, especially when overgrown plants require pruning and individuals are unwilling to discard cuttings due to their perception of plants as separate living entities.

The interaction practices with indoor plants that were most often mentioned included care, observation, tactile interaction, and recreation. Several additional practices were named as a continuation of PSRs with indoor plants, including giving names to plants, speaking to them, and making them a part of creative and study processes. Justification for these interaction practices included interest in the process, the need for a grounding routine, feelings of loneliness in new surroundings, and feelings of gratitude from plants. Tactile interaction and recreation, in particular, emphasize the sensory and emotional dimensions of human-plant relationships. Prior studies (Haller et al., 2019) have highlighted the role of tactile experiences—such as touching, repotting, and physically engaging with plants—in strengthening individuals’ bonds with them. This study supports those findings, as participants frequently described how handling plants provided a calming and meditative experience. Additionally, the recreational aspects of plant interactions—such as arranging them in aesthetically pleasing ways or using them as a backdrop for creative activities—further underscore their psychological significance (Clayton, 2007).

The findings regarding the practices people carry out in their relationships with plants appear to be in line with previous studies on people-plant interaction. Haller et al. (2019) created a spectrum of people-plant interaction that included physical exertion, peaceful abiding, and tactile immersion, and all of these components were found in the study, although some were more prominent than others. This study builds on Haller et al.’s framework by demonstrating that these types of interactions are not mutually exclusive but are instead interwoven into daily routines. For instance, participants who engaged in tactile immersion often simultaneously experienced peaceful abiding, using plant care as a meditative practice. These findings suggest that human-plant interactions fulfill multiple psychological functions, ranging from fostering relaxation to reinforcing personal identity (Beery and Jorgensen, 2018).

The narratives students used to legitimize the formation and maintenance of PSIs and PSRs with houseplants were defined by the ways students perceive their indoor plants. Five different types of relationships between people and their plants were identified. The intensity of the relationships varied across the different types, but in all of them, it was noted that relationships were diverse with fluctuating degrees of interest, depending on multiple factors. The main difference between the types of relationships was the perception of plant agency; for “owners” it was much lower than for “parents.” The narratives underlying the formation of people-plant relationships were reciprocity and homophily; participants formed the relationships based on their attitudes toward plants and their feelings of connectedness to them. The findings related to the para-social aspects of people-plant relationships are mostly in line with the literature on para-social phenomena. The homophily aspect of forming a meaningful and fulfilling relationship is shown to be quite influential because bonding with those viewed as similar to oneself serves a self-validation function and makes interactions smoother and more enjoyable (Fehr, 2008). Moreover, perceived reciprocity was an important aspect of people-nature relationships in the previous literature. Even though there is not enough research on this concept with indoor plants, the idea that nature and humans are linked in an equal relationship appeared in the history of many indigenous communities. Though it is not specifically common for modern-day people, the fact that such attitudes can be developed was stated (Cristancho and Vining, 2004).

Lastly, the factors that influence PSRs with indoor plants include living in a big city, moving to another city or country, the space in which the houseplants exist, and media consumption. Exposure, particularly the amount and quality of interactions with indoor plants, has been consistently identified as a significant factor in the formation and maintenance of PSRs, as shown by Hall and Davis (2017). They highlighted the importance of regular engagement and care activities in fostering emotional connections. The influence of exposure context, including living in a big city, physical space, and media consumption, has also been investigated in previous research. Urbanization and its impact on people’s relationship with nature have been examined by Reis et al. (2020), who found that individuals in urban environments may seek alternative ways to connect with nature, such as keeping indoor plants. This study contributes to the discourse by demonstrating that urban dwellers not only adopt plants to compensate for a lack of greenery but also develop parasocial bonds with them as a response to the transient and isolating nature of city life. The act of caring for plants provides a stabilizing routine, fostering a sense of continuity and control in an otherwise fast-paced environment. Our study echoes these findings, as participants discussed how their urban living context influenced their attitudes and practices with indoor plants.

### Implications

This study provides valuable insights into the psychological mechanisms underlying the formation of parasocial relationships with houseplants, demonstrating that these bonds are shaped by early exposure, urban living conditions, and social influences. The findings suggest that PSRs with plants share commonalities with those formed with other non-human entities, such as pets, fictional characters, and even deities, reinforcing the idea that human attachment extends beyond traditional interpersonal relationships.

From a psychological perspective, the study highlights how plant-related PSRs can serve as a coping mechanism for stress and loneliness, particularly in urban environments. This has significant implications for mental health research, as fostering connections with houseplants may represent a valuable, low-cost strategy for promoting well-being among individuals in high-density urban settings.

The findings also carry implications for urban planning and biophilic design. As cities become increasingly densely populated and green spaces diminish, encouraging plant ownership and integrating plant-friendly policies into residential and public spaces could enhance emotional well-being. Furthermore, the study suggests potential applications in educational and therapeutic contexts, where plant care could be incorporated into well-being programs or stress reduction interventions.

### Limitations of the study

This study is not without limitations. Firstly, the sample size in this study was relatively small, consisting of a specific group of participants. The sample also exhibited a bias toward residents of Moscow and large cities in general. It is important for future research to study the patterns of relationships between people and indoor plants in smaller towns and villages to enhance the generalizability of the findings. Considering this limitation, the generalization for other countries would be problematic because the general cohort of Russian students was quite narrow and it is important to recognize that different demographic groups or cultural contexts may have varying perceptions and experiences (Dworkin, 2012).

Secondly, the reliance on self-report measures and qualitative interviews introduces the possibility of response bias and subjectivity. Participants may have provided socially desirable responses or their perceptions may have been influenced by personal biases (Drisko, 2013). Additionally, it should be noted that only one researcher worked on the thesis, which could have unintentionally influenced the data collection, analysis, and interpretation of findings by the researcher’s personal prejudices, beliefs, and limited perspective (Jonsen and Jehn, 2009).

Furthermore, the cross-sectional nature of the study design limits our ability to establish causal relationships between the identified factors and the intensity of PSRs. Longitudinal studies or experimental designs could provide more robust evidence for understanding the temporal dynamics and causal mechanisms involved.

Lastly, it is important to acknowledge that PSRs with houseplants are complex and multifaceted phenomena. The current study focused on a limited set of factors influencing these relationships, and there may be additional variables and contextual factors that were not captured. Future research could explore other potential factors, such as individual personality traits, cultural influences, or specific plant attributes. A quantitative study using a larger sample could help with determining the exact factors involved and the nature of the relationship people have with their plants.

Despite these limitations, the findings of this study provide valuable insights into the formation and maintenance of PSRs with indoor plants among students. The limitations outlined here should be considered when interpreting the results and generalizing the findings to other populations and contexts.

## Data Availability

The raw data supporting the conclusions of this article will be made available by the authors, without undue reservation.
