# Supplementary material for: Application of the theory of para-social relationships for the analysis of people’s perceptions of indoor plants
Source: Front Psychol. 2025 Jun 18;16:1533128. doi: 10.3389/fpsyg.2025.1533128 (PMC12213490; doi:10.3389/fpsyg.2025.1533128)
Supplement: Supplementary file 1 [file Supplementary_file_1.docx]

**Appendices**

Appendix 1

***Interview Guide***

Hello, thank you so much for agreeing to be interviewed. Today we will be talking about indoor plants. Before we begin, may I record our conversation on a tape recorder? All data will be stored and analyzed in an anonymized and summarized form.

**Introduction:**

- So, to begin, please tell me about yourself. How old are you? What do you do? Where do you study? Where do you work?
- Now can you please tell me about your experience with indoor plants?
- What plants do you have now? You don't have to list the names, just describe them in a meaningful way. How many?
- Can you characterize them and your attitude toward them?
- How long have you had them?
- When did you start to be interested in plants, and when did you have your first indoor plants?
- How do you decide to buy an indoor plant?
- Any thoughts on which ones you would like to get in the future?

**Block 1: Exposition**

- How much time do you spend around indoor plants?
- Do you pay attention to plants when you are in the same room with them?
- Does your perception of colors differ from other objects in any space?
- Can you please describe how you generally interact with your indoor plants?
- Can you describe your routine with them? How often do you water, nourish, and re-pot?
- Do you think the amount of contact you have with your indoor plants affects your connection to them, your attachment to them?
  - Why or why not?
- Have you had the experience of moving and taking your plants with you?
  - If yes, how did you choose which ones to take?
  - If not, what was the experience of leaving them behind? Did you leave them in "good hands?"
- If you move in the future, will you take any houseplants with you?
- Who takes care of your houseplants while you are away?
  - Do you worry about your plants while you are away?

**Block 2: Para-Social Relationships with Indoor Plants**

- Can you please tell me if you have certain plants that are especially important to you?
- Have you ever felt a strong emotional connection with your plants, almost like another person?
- How would you generally describe your relationship with your indoor plants?
  - Are they more like your ward, are you neighbors, are you friends?
  - Is it more of a one-way or reciprocal relationship?
- Have you ever caught yourself talking to your plants as if they can hear you and even respond?
  - If so, how do you feel their responses manifest?
- Did you give them names?
- Do you feel that your indoor plants have a distinct personality or characteristics, or that they are more than just an object?

**Block 3: Homophilia**

- Are there any plants with which you associate yourself?
- Do you feel more connected to a houseplant that you see as being similar to you in some way (e.g., matching your personality, style, or interests)?
- Do you consider your love of indoor plants to be part of your identity?

**Block 4: Context of the exposure**

- Do you think living in a metropolitan area affects your relationship with indoor plants?
  - Why or why not?
  - Do you feel the need to spend time with your indoor plants after being in the city?
- Do you feel more rested after being in contact with your indoor plants?
  - If yes, please describe this experience.
- Do you follow accounts related to plant care or anything educational like that on social media?
  - Why or why not?
  - Has your consumption of any social media content, movies, or TV shows ever influenced your attitude toward plants?
- Do you use apps like Forrest, where you have to grow a tree for a certain period of time without being distracted by your phone?
  - What do you think about it? Would it help you to stay focused if you thought that if you picked up your phone, the plant would die?

**Conclusion:**

- Well, concluding our conversation, can you please tell me again your motivation for owning some number of indoor plants?
- Is there anything else that we haven't discussed yet that you would like to voice?

Appendix 2

## Table 1. List of Participants with Their Main Characteristics

| **Participant** | | | | | | **Interview** | |
| --- | --- | --- | --- | --- | --- | --- | --- |
| **Code** | **Gender** | **Age** | **Occupation** | **City** | **Living situation** | **Date** | **Duration** |
| 1LS | Female | 22 | Student | Moscow | Flat with partner | 02.04.2023 | 36 min. |
| 2VN | Female | 24 | Student, employed | Moscow | Flat with parents | 02.04.2023 | 46 min. |
| 3NV | Female | 23 | Student | Moscow | Dorms with roommates | 03.04.2023 | 79 min. |
| 4DH | Male | 19 | Student | Istanbul | Flat with partner | 04.04.2023 | 45 min. |
| 5NY | Female | 21 | Student | Moscow | Dorms with roommates | 06.04.2023 | 45 min. |
| 6KR | Female | 22 | Student, employed | Moscow | Flat alone | 08.04.2023 | 63 min. |
| 7AA | Female | 25 | Student | Moscow | Flat alone | 08.04.2023 | 52 min. |
| 8ND | Female | 21 | Student, employed | Moscow | Flat with roommates | 09.04.2023 | 48 min. |
| 9SG | Male | 21 | Student, employed | Moscow | Flat with parents | 09.04.2023 | 49 min. |
| 10MP | Female | 20 | Student, employed | Moscow | Dorms with roommates | 10.04.2023 | 48 min. |
| 11KR | Female | 28 | Student, employed | Kampala | Flat with partner | 11.04.2023 | 40 min. |
| 12KS | Female | 21 | Student, employed | Moscow | Flat with roommates | 11.04.2023 | 40 min. |
| 13DS | Female | 22 | Student | Moscow | Flat with parents | 02.05.2023 | 45 min. |
| 14KM | Female | 21 | Student | Brussels | Dorms alone | 06.05.2023 | 27 min. |
| 15MD | Male | 46 | Student, employed | Moscow | Flat with partner | 07.05.2023 | 28 min. |
